# Promoting Community Conversations About Research to End Suicide: learning and behavioural outcomes of a training-of-trainers model to facilitate grassroots community health education to address Indigenous youth suicide prevention

**DOI:** 10.1080/22423982.2017.1345277

**Published:** 2017-08-01

**Authors:** Lisa Wexler, Lucas Trout, Suzanne Rataj, Tanya Kirk, Roberta Moto, Diane McEachern

**Affiliations:** ^a^ Department of Health Promotion and Policy, School of Public Health and Health Sciences, University of Massachusetts, Amherst, MA, USA; ^b^ Wellness Program, Maniilaq Association, Kotzebue, AK, USA; ^c^ College of Rural Development, University of Alaska Fairbanks, Yukon-Kuskokwim Campus, Bethel, AK, USA

**Keywords:** Suicide prevention, training of trainers, Indigenous, community health education, Alaska Native, feasibility study

## Abstract

Alaska Native (AN) youth suicide remains a substantial and recalcitrant health disparity, especially in rural/remote communities. Promoting Community Conversations About Research to End Suicide (PC CARES) is a community health intervention that responds to the need for culturally responsive and evidence-supported prevention practice, using a grassroots approach to spark multilevel and community-based efforts for suicide prevention. This paper describes theoretical and practical considerations of the approach, and assesses the feasibility and preliminary learning and behavioural outcomes of the training-of-trainers model. It details the training of a first cohort of intervention facilitators in Northwest Alaska (NWA). Thirty-two people from 11 NWA village communities completed the PC CARES facilitator training, preparing them to implement the intervention in their home communities. Facilitator pre-post surveys focused on readiness to facilitate, a group quiz assessed participants’ understanding of relevant research evidence, and practice facilitation exercises demonstrated competency. Curriculum fidelity and accuracy scores were calculated using audio recordings from learning circles conducted by facilitators in their home communities. Facilitator reflections describe the successes of the model and identify several areas for improvement. As of March 2017, 20 of the 32 trained facilitators in 10 of the 11 participating villages have hosted 54 LCs, with a total of 309 unique community members. Coding of these LCs by 2 independent raters indicate acceptable levels of fidelity and accurate dissemination of research evidence by facilitators. Facilitator reflections were positive overall, suggesting PC CARES is feasible, acceptable and potentially impactful as a way to translate research to practice in under-resourced, rural AN communities. PC CARES represents a practical community education and mobilisation approach to Indigenous youth suicide prevention that displays preliminary success in learning and behavioural outcomes of local facilitators.

## Introduction

Suicide among Alaska Native (AN) and other circumpolar Indigenous youth represents a significant health disparity that poses a particular challenge in rural and remote communities. AN villages have limited behavioural health care infrastructure [1–3], and require that mental health care providers have local and cultural knowledge context to navigate effectively [4]. In these settings, many service providers are non-Native, and the measures taken in acute suicide events may cause unintended cultural harms as individual and family liberties are curtailed to promote safety [5,6]. To reduce reliance on crisis-oriented suicide care, a coordinated and sustained community-based early response is warranted. Such an approach draws on local knowledge, reflects social and cultural realities, and builds upon durable local systems of care [4–6]. These kinds of community-based preventative strategies require developing local capacity in under-resourced communities.

Relying heavily on professional mental health services to prevent suicide in rural and remote AN communities has often proven ineffectual [1–3]. In Northwest Alaska, only 8% of those who die by suicide and 32% of those who attempt have utilised mental health services over their lifetimes before a first attempt [1]. There are, however, documented risk factors that indicate a wider system of care – in addition to mental health services – be involved in suicide prevention. AN youth are likely to engage in dangerous activities [1], receive health care for alcohol-related injuries [3], drop out or get in trouble at school [1,3], and interface with law enforcement [1,3] in the months before exhibiting suicidal behaviour. These adverse events are strong indicators of suicide risk, and suggest opportunities for structuring additional community-wide prevention efforts in which mental health and medical workers, law enforcement, school personnel, tribal leaders and others work together to increase safety and support before a person becomes suicidal [1]. However, many people in key positions lack sufficient training to recognise suicide risk or to support vulnerable persons, and rarely is there village-wide collaboration and mobilisation before an acute event [1–3,7].

In addition, most suicide prevention trainings target gatekeepers in traditional clinical roles, who tend to be community “outsiders” in rural Alaska. This approach can limit the diffusion of information and resources to the rest of the community where they are most needed *and* most deployable. Many community health workers, religious leaders, social service administrators and tribal office workers are local people who are well positioned to make use of suicide prevention and intervention tools in their daily lives [3,5,6]. Mobilising such an array of local service providers can spur multilevel suicide prevention efforts that build on and extend local support systems – including those outside the bounds and scope of clinical intervention [4,6]. Such community-based approaches can develop village-based capacity and facilitate community-wide collaboration to address the vulnerabilities of persons – and communities – before individuals become suicidal. This kind of wide-ranging and multi-sector preventative programming has not been widely utilised in low-resourced rural and remote AN communities, but such an approach could leverage limited resources and have a significant impact.

### Intervention description

Promoting Community Conversations About Research to End Suicide (PC CARES) is a community health education intervention that addresses the need for culturally responsive, multi-sector, early suicide prevention practice that builds on and extends community infrastructure and support. Facilitated by local Indigenous leaders, PC CARES brings together community and family members, village-based paraprofessionals and regional health workers to attend 9 3-hour learning circles (LCs) over the course of a year. These LCs highlight research on suicide prevention and wellness that is relevant to circumpolar Indigenous communities in order to spark community discussions and personal storytelling that links the research to people’s experiences and knowledge.

**Figure 1.**
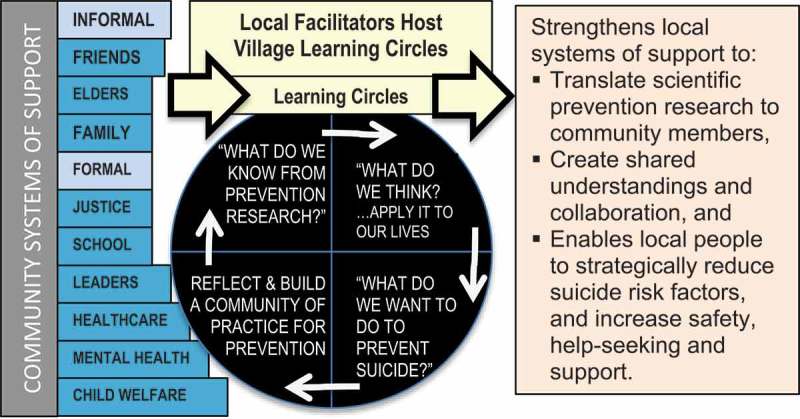
Conceptual model: Promoting Community Conversations About Research to End Suicide.

In LCs, local facilitators share information so participants learn “what works for prevention”, such as restriction of lethal means [8,9], and then get the opportunity to talk about “what they think” about it. Discussions could include how to increase safe gun storage options in village households. Participants then discuss “what they want to do” in their jobs, families and communities to put that information to use; for example, making guns locks available through village clinics and raising awareness in their families about their potential benefit (see Figure 1).

As opposed to didactic education models, LCs aim to be inclusive, engaging and culturally respectful to increase the likelihood that sessions are personally meaningful and foster durable learning and action [10]. The pervasive didactic suicide prevention educational models, such as gatekeeper training, have had disappointing learning and behavioural outcomes in Indigenous communities [5,11], indicating a need for innovation.

PC CARES builds on the conviction that the solutions to complex problems like AN youth suicide will build on research but be determined by affected, invested and involved community members themselves. By sharing research evidence with community members who are cultural and community experts, PC CARES offers a way to build mutual understandings of “best practices” among those in key roles to strategically prevent suicide. This public health approach shows promise [12] and has been called for by suicide experts generally [13], and specifically for Indigenous people [14,15]. Such ongoing community learning processes have been effective in other low-resource communities [16–19].

### Curriculum development

To develop the PC CARES curriculum, a working group led by PI Wexler of academics, mental health practitioners and rural AN community members discussed and identified a variety of research evidence relevant to suicide prevention. This content came from local studies, other Indigenous communities, and the prevention literature at large. The information was prioritised for its practical application for community members of all sorts, and 8 distinct areas of content were integrated into 8 LCs, with the final ninth LC a review. Nine LCs seemed feasible for each village to hold over the course of a year. The content of these sessions includes community-level conditions (#1,4,6) [20–22], evidence-based approaches (#5,6,7) [23–25], risk (#1,3,8) [1–3] and protective factors (#2,4,5) [18,19,26,27] that can reduce suicide risk and promote wellbeing. Teaching tools vary and include charts, infographics, short films and case studies. To aid local facilitators, each LC follows the same structure: What do we know? What do we think? and What do we want to do? (see Box 1).

**Table:** Box 1. PC CARES content, sharing method and learning circle format content and sharing method.

(LC 1)*Film* re: Historical trauma, culture suicide (LC 2)*Chart*: Adults roles in youth suicide prevention (LC 3)*Figure*: Seasonality and Alaska Native youth suicide (LC 4)*Charts*: community protective factors (LC 5)*Film*: Supportive counseling as prevention (LC 6)*Image*: Restricting lethal means (LC 7)*Case study*: Follow up after a suicide attempt (LC 8)*Chart*: Postvention: What to do /not to do to (LC 9)*Posters*: Review all and plan moving forward **Format of the 3-Hour Monthly Learning Circle Format**: Create a hospitable space (safe, private) where traditional practices are respected (e.g. Elders, circle seating, food) 1. Start with prayer led by an Elder from the community 2. Establish agreements for how to work together & remind participants about safely talking about suicide (posters) 3. Reflections about last meeting – reporting of actions taken (“small wins”) 4. Go over the purpose of PC CARES and of particular session • WHAT DO WE KNOW? “Bite-size” information from research (5–10 min) • WHAT DO WE THINK? Storytelling to consider the research (45–60 min) • WHAT DO WE WANT TO DO? Apply the research (20–30 minutes) 5. Check-out – Circle so that everyone gets a chance to speak 6. End with a prayer led by an Elder from the community

### Curriculum and materials

PC CARES materials include a detailed, step-by-step facilitators’ guide, and a toolkit that includes all the materials facilitators need to implement PC CARES (a portable file box with handouts, DVDs, markers, surveys, audio recorder with thumb drives, and portfolio carrier with posters). A programme website (www.pc-cares.org) offered facilitators and interested participants access to the curriculum, facilitators’ guide and the research papers supporting each LC.

## Methods

We employed several methods to recruit facilitators and track their readiness to facilitate PC CARES in their home villages. Procedures to audio record LCs offered us a way to document fidelity to the curriculum and interpretation of research evidence during the community LCs. Local facilitator reflections about the model’s strengths and areas for improvement were collected at the mid-point and end of the implementation period. All research was approved by the institutional review board of the [host institution], and conducted in partnership with the tribal health and social services organisation serving the region.

### Recruitment and initial piloting

To ensure that the content and the methods used in the LCs were acceptable to local participants and potential facilitators, we piloted several LCs in 6 villages across Northwest Alaska in August and September 2015. When piloting LCs in these communities, members of the research team solicited feedback, spread awareness of PC CARES and tried to generate interest in becoming a facilitator. Key leaders and participants were invited to become facilitators or to suggest and recruit other community members who were either in positions that supported their role of facilitator, such as village counsellors or resource specialists, or who had facilitation skills and interest in preventing suicide.

### Facilitator training

#### Facilitator readiness surveys

Pre and post-training surveys were used to assess attendants’ readiness to facilitate PC CARES LCs in their villages and to assess attendants’ satisfaction with the training they received over the course of the week. Participants were asked to use pen and paper to fill out 19 readiness questions on the first morning of the training (before any training sessions had begun) and then again on the last day of the training after training was complete. Readiness questions asked participants to rank their readiness to facilitate PC CARES sessions in their village using a 5-point Likert scale that ranged from “Strongly Disagree” to “Strongly Agree”. Data was entered into an Excel spreadsheet coded with “Strongly Disagree” having a value of 1 and “Strongly Agree” having a value of 5. Mean scores were calculated for each question and the Wilcoxon signed rank test was run using STATA 14 to determine the statistical significance of the change from paired surveys of the mean of the pre-test to the mean of the post test for facilitator readiness.

Facilitator trainees were also invited to fill out a *****satisfaction survey***** that was given after attending the first LC of the training and again after participating in all of the LCs and facilitating one at the end of the week’s training. This 25–item, 5-point Likert scale questionnaire asked facilitators to reflect on the usefulness of PC CARES and their satisfaction with it. Data was coded to numeric values and mean scores were taken for each item. The Wilcoxon signed rank test was run using STATA 14 to determine the statistical significance of the change from the mean of the survey done after 1 LC to the mean of the post-survey, completed after doing all 9 LCs.

#### Facilitation demonstration

Each village facilitation team had an opportunity to facilitate one LC with support from the trainers and their peers. At the training, facilitators were given time to practise until each member felt comfortable with the LC content and process. Then, they led the session in a role play and received comments from participants and trainers about what they did well, and could have done better.

#### Group content quiz

At the end of the training, a group true/false content quiz tracked each participant’s understanding of the research evidence presented in each of the LCs (see Box 1). In order to show their knowledge as individuals in a fun, nonthreatening way, participants were seated in rows facing the front of the room and given large placards to indicate “True” or “False” to 16 questions pertaining to the distinct subject matter shared in the LCs. These questions were read aloud by a trainer and shown in written form to the group. For example, one item stated, “Research shows that youth suicidal behaviour is more likely to happen in the winter”. To assess the accuracy of the statement, each participant responds by holding up either the “True” or “False” placard. Participants were asked to refrain from showing their signs to each other, in order to minimise group-influenced responses. Two members of the research team facing the room documented the number of incorrect answers to the 16 true-false questions. Respondent answers were recorded, and after the group quiz, all questions were discussed to clarify the meaning of the correct answers and to identify areas of confusion if participants did not answer 100% correctly.

#### *PC CARES implementation:* curriculum fidelity and accuracy of research interpretation

As local facilitators host LCs in villages across the participating remote and rural region of Alaska, we track adherence to the curriculum and structured dialogue procedures by audio recording and transcribing sessions, if all participants agree to this process. Facilitators put these recordings on a thumb drive and mail them to the research team. Once received, the audio recordings are transcribed verbatim.

Two independent raters independently coded LC transcripts to assess whether facilitators followed the format outlined in the Facilitator’s Guide, and interpreted the research information accurately. The information accuracy scale rates both the information presented by facilitators about the research evidence shared in that circle. A 3-point scale was used, with a score of 1 indicating that research evidence was interpreted accurately, leading to conclusions consistent with its intent; a score of 2 indicating that there are no direct misinterpretations of research, but the conversation focused primarily on issues of wellness or suicide prevention not directly related to (or evidencing understanding of) the research presented in the LC; and a score of 3 indicating that research evidence was interpreted inaccurately, leading to conclusions inconsistent with its intent and/or not contributing to productive community conversations. A narrative rationale is provided for each score (for example, documenting the manner in which research evidence was misinterpreted), and scores are averaged across both reviewers’ ratings.

Fidelity to the PC CARES curriculum is assessed across each of the 6 standard elements of each LC [1]: agreements/safe talk [2], small wins [3], the LC activity [4], what we know [5], what we think, and [6] what we want to do. Each dimension gets 0–1 for procedural components (i.e. presenting specific data, giving clear instructions to the group) that are present [1], absent (0), or not captured (NA) on the recording when the audio clearly misses some of the LC. Scores across LCs are averaged across the 2 raters.

### Facilitator feedback

At the mid-point and end of the PC CARES year-long intervention pilot, we brought facilitators together to document their reflections on PC CARES and to integrate their ideas for improvement into the model. Session notes documented key ideas shared in these sessions. The research team identified main themes in these notes and shared them with facilitators to be sure they adequately captured key ideas.

## Results

### Facilitator training

Local leaders who were familiar with PC CARES recruited 32 people from 11 regional villages to participate in a 40-hour facilitator training, on the basis of interest as well as formal or informal leadership/care roles in their communities. Many who participated were employees of the for-profit tribal regional corporation, which was very supportive of PC CARES because of its commitment to village wellness. An additional 4 people from other parts of the state attended the training due to their interest in the model.

**Figure 2.**
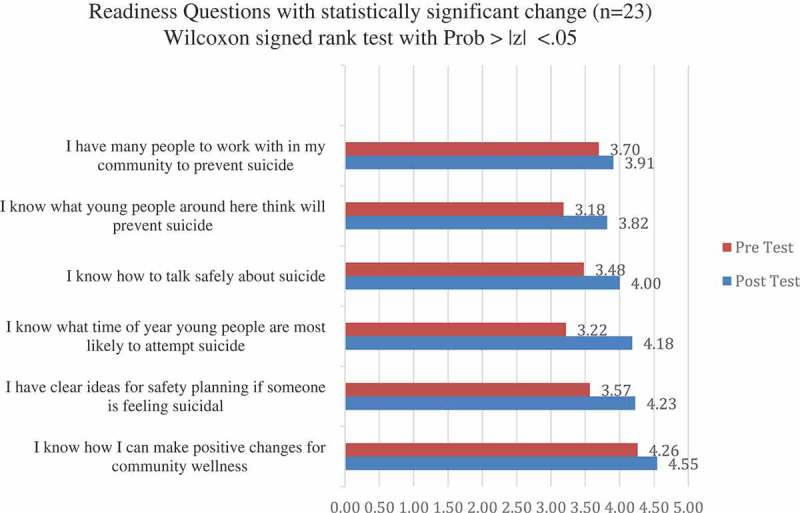
Readiness of facilitators to support suicide prevention in their communities before (pre) and after (post) attending the week-long facilitator training.

Due to an unanticipated flight delay on the first day and a scheduling issue on the last, only 23 of the NWA facilitators in the training (out of 32) completed both a pre- and post facilitator readiness survey. Among the 19 questions measuring readiness, 15 moved in the predicted positive direction, indicating participants felt more ready to facilitate at the end of the training when compared with the beginning, and 6 were statistically significant as indicated by the Wilcoxon signed rank test. Results demonstrate increases in facilitators’ readiness to engage in prevention activities in their communities and in perceived personal and community-level capacity for suicide prevention. Survey items showing statistically significant change are in Figure 2.

**Figure 3.**
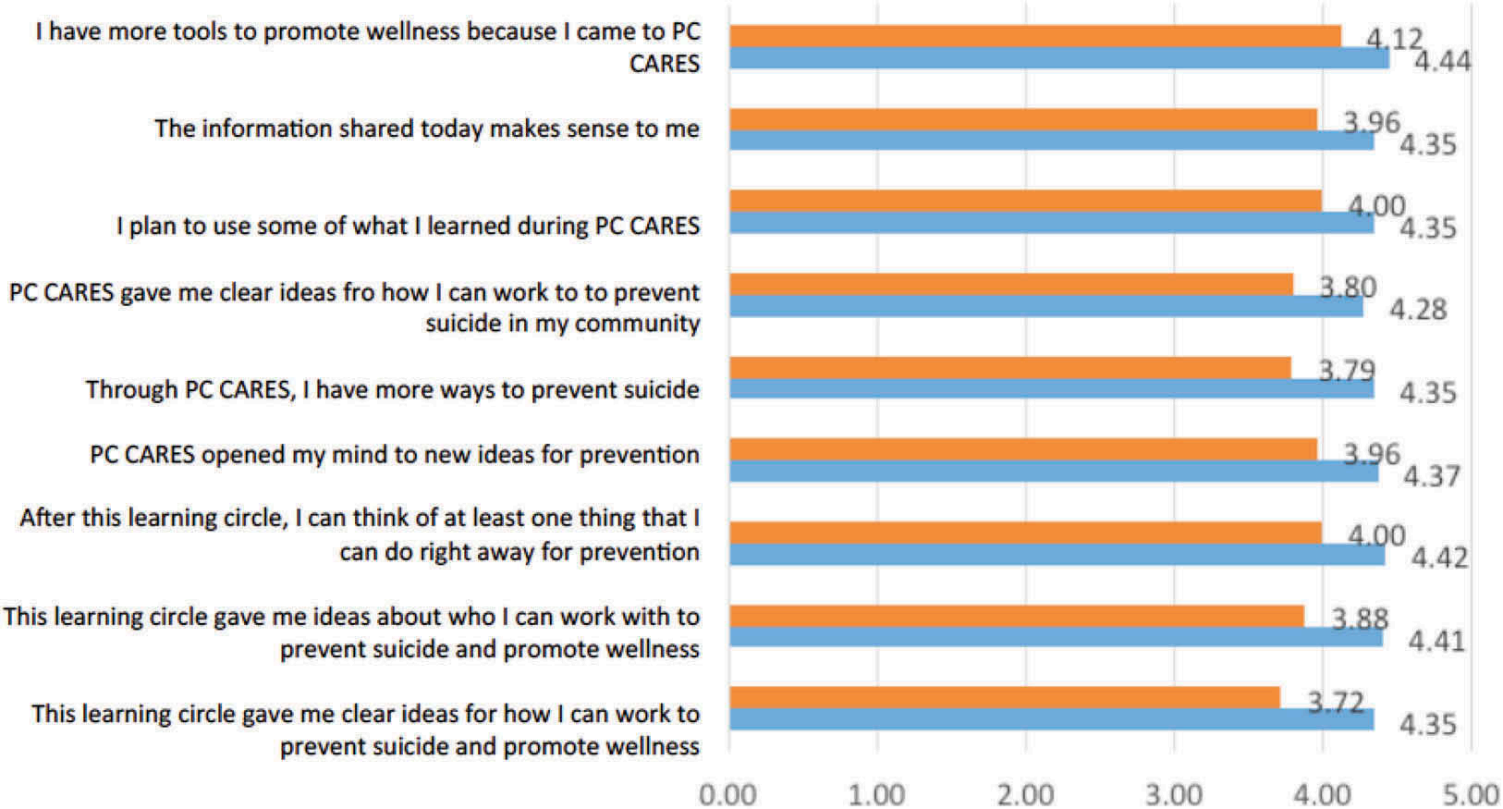
Facilitator satisfaction with PC CARES (after participating in LC1 compared with after participating in all 9 LCs over the course of the week-long training).

Satisfaction with the PC CARES LC approach was assessed after facilitators attended the first LC and then again at the end of the week-long training, after completing all the LCs and facilitating one. Among the 25 questions measuring satisfaction, the mean scores of 22 questions moved in an expected positive direction, and of those 9 were statistically significant. They are shown in Figure 3.

In addition, based on data from the post-survey given at the training, 90% of facilitators found PC CARES to be a culturally responsive way to engage community members in suicide prevention efforts, and the majority (20 of 32) of facilitators initiated LCs in their villages within 3 months of the training.

#### Facilitation demonstration

All trainees worked in teams from their home village to facilitate a LC during the training. These teams reflected on and received feedback on “what worked” and “how to improve” from the trainers and by their peers. All teams followed the facilitator guide proficiently, with guidance mainly focused on facilitation skills (e.g. speaking loudly, giving directions before handing out materials).

#### Group quiz to assess knowledge about suicide prevention

Answers in the group true-false quiz, 92% of the trainees answered 11 of the 16 questions correctly, and 70% answered the other 5 questions correctly. Trainees told us that these 5 questions had ambiguous wording. For instance, one item stated, “After someone has attempted suicide, he or she probably wants to be left alone.” This statement was considered confusing because some people felt that such experiences are situational, relational and contingent. If, however the phrasing of “research shows that…” begins the statement, participants felt more certain about how to assess the item (false) because it is not about a specific and personal case but rather about the research evidence shared in LC 7. We modified all the items to reflect that they refer to research rather than contingent and personal experiences, and re-asked the 5 questions which were answered 100% correctly.

#### Programme implementation

As of March 2017, 20 of the 32 trained facilitators in 10 of the 11 participating villages have hosted at least 2 PC CARES LCs in their home community. All but 3 who did not follow through left or changed their jobs, and/or moved from the village. The active PC CARES facilitators hosted a total of 54 LCs, with a total of 309 unique community members attending (in a region with a total population of approximately 8000) (See Table 1). Average attendance in each LC was close to 9 people per LC. The data collection procedures for village LCs have proven feasible. In the 54 LCs done to date, facilitators have consistently informed participants about the research, collected attendance and survey data, and in 53 of the sessions participants gave permission for audio recording.

Table 2 provides a summary of the data collection and analysis described above. Table 1 shows village participation in the region.

**Table 1.** Number of villages hosting each learning circle & participants.

	Number of villageshosting each LC	Number of attendees combined
LC1	10	160
LC2	10	83
LC3	9	76
LC4	7	54
LC5	5	39
LC6	4	21
LC7	4	13
LC8	3	9
LC9	2	10
**Totals**	**54 LCs done in Villages**	**465 (not unique)**

**Table 2.** Data collection and analysis.

Measure description	When given	Examination of findings
Facilitator readiness: 19-item, 5-point Likert scale of agreement	Before and after week-long TOF	Paired pre-post means compared using Wilcoxon signed rank test with STATA 14 (n=23)
Facilitator satisfaction: 25-item, 5-point Likert scale of agreement	After facilitators participated in LC1, and after attending all LCs in the TOF	All surveys for each time point (after LC1 and after doing 9 LCs in the TOF) are combined to find means for each item and compared using Wilcoxon signed rank test with STATA 14
Demonstration of facilitation	During the TOF, each facilitation team hosted 1 LC	Trainers participated in the LC facilitated by trainees, and offered feedback about their session: what went well and could be improved
Group Quiz about LC research content with individuals answering T/F questions about the research content of LCs	End of TOF	All responses recorded by team, and correct answers are discussed with rationale. Areas of confusion are clarified in a group setting
Implementation or process tracking in villages		
Accuracy in interpreting research evidence presented in LCs	Audio recording of LCs taking place in villages	3-point scale used to assess the degree of accuracy: 1=accurate, aligned with intent, to 3=inaccurate
Fidelity checking: documenting the extent to which local facilitators adhered to Facilitator Guide	Audio recording of LCs taking place in villages	Transcribed and coded by 2 independent reviewers: 1–0 for following the procedures on 6 LC elements, averaged for each session and across all sessions
Facilitator feedback in group discussions	Facilitator meetings midway and after doing PC CARES in their villages	Notes taken during the 2 in-person meetings in April 2016 and January 2017 were analysed for basic themes and shared back with facilitators for their approval

#### Curriculum fidelity and information accuracy

At present, 65% of the LCs have been transcribed and coded by 2 independent raters. Results show that there is a high level of accuracy, with an average score of 1.21. This score means that, for the most part, the research shared in the LC was interpreted correctly, leading to community conversations in line with the learning objectives. We consider scores under 1.5 to be of adequate accuracy. Importantly, there were no cases of facilitators or participants having inaccurate interpretations of research evidence. The majority of facilitators demonstrate fidelity to the PC CARES curriculum. Two independent coders from the research team generated an average fidelity score of .79, indicating acceptable adherence to our curriculum. This score means that key LC elements were covered adequately by local facilitators who followed the PC CARES Facilitators’ Guide with only small variations. The inter-rater reliability on fidelity measures was 85.3%.

#### Facilitator reflections

Since initiating PC CARES in December 2015, the research team brought active facilitators together twice to reflect on the feasibility and value of the model, and to identify and address areas for improvement. At the first 2-day meeting at the mid-point of the intervention (April 2016), 18 of the 20 active facilitators came together in the regional hub community to share their experiences with each other and the developers. The main facilitator challenges related to logistics (finding quiet space, getting snacks, having a working TV to show DVDs), recruitment and the difficulty of getting a consistent group of attendees, and facilitating discussion when there were very talkative participants or persons in attendance who they did not feel should not be re-directed, Elders for instance.

The preliminary successes of the model at mid-point focus on people coming together to learn and solve problems. Facilitators’ general comments included sentiments found in the following 2 quotes: “I was really happy to see Inupiaq people in a shared space where they can talk about their healing and how we are going to move forward”; “It’s great to see people in the villages being in charge of their own. It’s a great model for us to work with. It has a lot of cultural pieces to it when we do our work and want to get well”.

Approximately a year after initiating PC CARES, 12 active facilitators were brought together again to reflect on the whole intervention, and provide ideas for increasing successful implementation in the future. They said the materials were easy to work with and that some of the logistical issues with snacks, DVD equipment and managing the materials of LCs got easier to manage over time. Facilitators shared a variety of preferences for different kinds of content. Some facilitators felt like starting with a positive content (i.e. supportive counselling) rather than historical trauma would be better; others had general preference for pictures, easy-to-decipher graphs, rather than case studies and films. Others preferred the short films. Facilitators reflected their general belief that some content was more interesting to community members than others, and those sessions were therefore better attended. Most facilitators also thought the different activities in each session were helpful within a set structure to keep the LC both predictable and interesting.

Suggestions for improvement included reducing the number of LCs and including more diverse content in each LC to appeal to more people, having LCs more frequently than once per month, and giving facilitators options for content, depending on the needs of their village at different times. The biggest shared challenge across villages was maintaining consistent attendance over time, and suggestions for addressing this included offering continuing education credit (CEUs) for community workers, and more personal recruiting.

## Discussion

The results from this pilot contribute to our understanding of the feasibility of using a training-of-trainer model to share suicide prevention research with community members. An Institute of Medicine workshop focusing on reducing health disparities in 2012 reported a clear need for more translational research that will bring the existing knowledge base into action [28]. Too often, research focuses on developing and studying interventions within ideal circumstances, which are difficult to replicate in resource-stretched community settings [29]. Feasibility is thus a primary concern that must be addressed for interventions to have their intended impact. With practical implementation concerns, this study considers and documents the feasibility of a community education model done by local facilitators in a rural and remote AN region.

Aimed at building the capacity for evidence-based and self-determined suicide prevention in under-resourced communities, PC CARES relies on local facilitators to do much of the vital work. Because the model depends on local AN volunteers with limited formal education to implement PC CARES, the curriculum and materials need to be user friendly, meaningful within the local and cultural context so facilitators do it, and not too time consuming to implement. To implement village LCs, facilitators must be trained enough to feel sufficiently prepared and supported in doing a good job [30]. The training of facilitators is more likely to be successful if it allows for a diversity of learners and perspectives, and integrates practice into training to enhance the skills of participants [31].

Results from the first pilot of PC CARES indicate that after 1 week of training, facilitators increased their readiness, meaning their confidence and endorsement of the model as a way to initiate prevention activities in their community. Their satisfaction with the model increased after doing the first LC when compared with their perspectives after doing all 9 in the training of facilitators (TOF). Each facilitation team demonstrated their ability to facilitate a LC, documenting an important performance result of the training [32] that has rarely been documented in other studies [33]. In addition, the facilitators’ group quiz seemed to be an acceptable, even fun, way to assess whether they adequately understood the research evidence shared in the LCs.

These factors increased facilitators’ confidence and commitment to facilitate PC CARES, no small feat when considering the sometimes-new leadership role facilitation required. One facilitator said after attending the week-long training to prepare her, “Before this week, I thought this [being a facilitator of PC CARES] was going to be too much on my plate; during this week, I learned that everyone in the community needs to participate in saving lives, and now I think I am in the right place as a facilitator of PC CARES.” Research indicates that facilitator confidence, belief in the practice, support and limited time commitment increases the likelihood that people trained as trainers will implement the training [33]. The majority of those trained as PC CARES facilitators did follow through and offer at least 2 LCs in their home community. This outcome adds to the scant literature on the percentage of lay people trained as facilitators go on to offer the programme [34].

Few studies have tracked the implementation outcomes of training-of-trainers models [33]. Here, we document the fidelity and accuracy scores from village LCs facilitated by those trained. The results are acceptable, and higher than has been found in other trainings of trainers for suicide prevention [31]. The high fidelity scores coincide with facilitator feedback that suggests the facilitation materials with step-by-step instructions were easy to use.

The study also identified some areas for improvement. Reflection from the facilitators, variability in village implementation (some villages did 2 LCs while others did all 9, see Table 2) and inconsistent attendance over time suggests a need to make some changes to the intervention. First, when recruiting facilitators, it is important to identify persons who are planning on staying in their job or home community for at least another year. To encourage more consistent village completion of all the LCs, content can be combined so that the same amount of material can be covered in fewer LCs. To increase facilitator self-determination and perhaps confidence, they can be given a menu of LC options which they can offer according to their perceptions of local interest and usefulness. This change addresses the different opinions expressed by facilitators about the order of the content and interests of community members at different times. Facilitators believe that hosting LCs weekly or biweekly LC will maintain momentum of participants and lead to more consistent attendance for a shorter period of time. Monthly sessions over approximately a year were too difficult to coordinate around annual subsistence activities and travel schedules, and were perhaps too spread out to maintain commitment. In addition, formally offering CEUs to behavioural health and health care providers who attend the majority of LCs may also encourage consistent attendance.

## Limitations

The results reported in this study have several limitations. The sample size is small with only 32 trainees from the participating region, and reduced further by the unanticipated travel and scheduling issues that led to only 23 paired pre-post readiness surveys and 31 satisfaction surveys completed. Although the audio recording of village LCs offers some insight into the implementation of the programme in the communities, the transcripts do not show nonverbal interactions that could impact facilitation. The facilitator feedback gathered at 2 time points is likely to be positively influenced by interactions with the research team, who both collected the information and developed the intervention. Lastly, the feasibility study did just that: assessed the learning and behavioural outcomes of the training-of-trainers model for local facilitators in a rural and remote region of Alaska. It did not assess community learning outcomes of the village PC CARES sessions, the impact the LCs may have had on participants’ preventative activities, nor did it document the effect of the intervention on suicidal behaviours, the ultimate outcome. These impacts represent an important next step in the evaluation of the intervention.

## Conclusions

Our feasibility study of PC CARES demonstrates practical success in training local volunteers to facilitate LCs in their home communities. The approach aims to share “what we know” from suicide prevention research with community members so that they can discuss “what they think” about it as they apply it to their local context, and figure out “what they want to do” to promote health and prevent suicide in their jobs and lives. Our focus on learning and behavioural outcomes from our TOF gives us reason for optimism about the scalability of the model. Measures captured learning outcomes of the TOF by increasing facilitators’ readiness to facilitate LCs, and fostering increased appreciation for the model. By recording and coding transcripts from village sessions facilitated by those trained, we demonstrate acceptable fidelity to the model and accuracy in the research disseminated. This result suggests that the week-long facilitator training and materials provided adequate preparation for lay volunteers to implement the programme. Facilitator reflections about the model were also positive, giving a sense that PC CARES is a culturally acceptable, empowering and potentially impactful way to share prevention research with community members who can put it to strategic use. Adjustments to the model address the lack of consistent attendance over time. In conclusion, the promise of PC CARES is that it offers rural and remote Indigenous communities a practical and scalable method for translating research evidence into community-driven, culturally responsive suicide prevention practice.
